# Effect of two counseling interventions on self-reported alcohol consumption, alcohol biomarker phosphatidylethanol (PEth), and viral suppression among persons living with HIV (PWH) with unhealthy alcohol use in Uganda: A randomized controlled trial

**DOI:** 10.1016/j.drugalcdep.2023.109783

**Published:** 2023-01-21

**Authors:** Judith A. Hahn, Robin Fatch, Nneka I. Emenyonu, Naomi Sanyu, Anita Katusiime, Barry Levine, W. John Boscardin, Geetanjali Chander, Heidi Hutton, Carol S. Camlin, Sarah E. Woolf-King, Winnie R. Muyindike

**Affiliations:** a Department of Medicine, University of California, San Francisco, CA, USA; b Faculty of Medicine, Mbarara University of Science and Technology, Mbarara, Uganda; c Department of Computer Science, San Francisco State University, San Francisco, CA, USA; d Department of Medicine, University of Washington, Seattle, WA, USA; e School of Medicine, Johns Hopkins University, Baltimore, MD, USA; f Department of Obstetrics, Gynecology, and Reproductive Sciences, University of California, San Francisco, CA, USA; g College of Arts & Sciences, Syracuse University, Syracuse, NY, USA

**Keywords:** Brief alcohol intervention, Unhealthy drinking, Phosphatidylethanol, Social desirability bias, HIV, Sub-Saharan Africa

## Abstract

**Purpose:**

To test the efficacy of two interventions to reduce alcohol use and increase viral suppression compared to a control in persons with HIV (PWH).

**Methods:**

In a three-arm (1:1:1) randomized controlled trial (N = 269), we compared in-person counselling (45–70 minutes, two sessions over three months) with interim monthly booster phone calls (live call arm) or twice-weekly automated booster sessions (technology arm) to a brief advice control arm. We enrolled PWH self-reporting unhealthy alcohol use (Alcohol Use Disorders Identification Test – Consumption, prior three months, women ≥3, men ≥4). Primary outcomes were number of self-reported drinking days (NDD) in the prior 21 and biomarker phosphatidylethanol (PEth) at six and nine months and viral suppression (<40 copies/mL) at nine months; we adjusted for sex and baseline outcomes.

**Results:**

At baseline, mean 21-day NDDs were 9.4 (95 % CI: 9.1–9.8), mean PEth was 407.8 ng/mL (95 % CI: 340.7–474.8), and 89.2 % were virally suppressed. At follow-up, there were significant reductions in mean NDDs for the live call versus control arm (3.5, 95 % CI:2.1–4.9, p < 0.001) and for the technology versus control arm (3.6, 95 % CI: 2.2–5.1, p < 0.001). The mean PEth differences compared to the control arm were not significant, i.e. 36.4 ng/mL (95 % CI: −117.5 to 190.3, p = 0.643) for the live call and −30.9 ng/mL (95 % CI: −194.8 to 132.9, p = 0.711) for the technology arm. Nine-month viral suppression compared to the control was similar in the live call and in the technology arm.

**Conclusion:**

Intervention effects were found on self-reported NDD but not PEth or viral suppression, suggesting no treatment effect.

(NCT #03928418)

## 1. Introduction

Alcohol consumption is a critical driver of poor HIV outcomes,especially in sub-Saharan Africa (SSA), where both are extremely common (Hahn et al., 2011). Unhealthy alcohol use, defined as drinking above recommended limits, has been associated with reduced antiretroviral adherence, decreased HIV suppression, and increased mortality among those with HIV (Williams et al., 2016). Reducing unhealthy alcohol use is likely to improve HIV outcomes (Satre et al., 2020), and is therefore a high health priority worldwide.

The vast majority of PWH live in low-income settings, thus low-cost alcohol interventions, such as brief counselling-based interventions, are needed. A meta-analysis of alcohol counselling interventions conducted among PWH worldwide, though primarily in high income countries, showed a small but significant effect on reducing total amount of alcohol consumed and on HIV viral suppression (Scott-Sheldon et al., 2017). However, a more recent systematic review found no consistent effect of counselling on these outcomes (Madhombiro et al., 2019). Few brief alcohol interventions have been examined in sub-Saharan Africa (Francis et al., 2019), thus there is an urgent need to investigate brief alcohol interventions for PWH in such settings.

It is also unknown how best to deliver low-cost alcohol interventions in low-income settings. Multi-session interventions that combine inperson visits with booster phone calls to reinforce the in-person counseling have shown efficacy (Chander et al., 2015; Fleming et al., 1997; Go et al., 2020). Because cell phone use in Uganda is high, phone-based booster sessions to supplement in-person sessions may be feasible. In addition, automated cell phone-based booster sessions, previously successful in improving health behaviors (Hall et al., 2015), can be conducted via systems such as two-way Short Message Service (SMS, i.e. text messaging) or Interactive Voice Response (IVR) that allow for brief interactive, two-way sessions. However, the uptake, acceptability, cost, and efficacy of live and tech-based booster calls for interventions for reducing alcohol use and improving HIV outcomes in SSA is not known.

Lastly, the primary outcomes for all prior brief intervention trials were measured by self-report, which may be subject to social desirability bias because participants who are counselled to reduce alcohol use may be especially vulnerable to under-reporting (McCambridge and Saitz, 2017). There is a need to employ objective measurement of alcohol intervention effects; the use of biomarkers such as phosphatidylethanol (PEth) has been suggested (Madhombiro et al., 2019).

The primary aim was to conduct a three-arm randomized controlled trial (RCT) to examine the efficacy of a brief counselling-based intervention among PWH with unhealthy alcohol use in southwest Uganda. The intervention included two in-person sessions and booster sessions delivered by either monthly live counsellor phone calls or by twice-weekly automated two-way messaging. We compared each to a standard of care control at follow-up (six and nine months after baseline). Our primary outcomes were number of drinking days by self-report and PEth, and HIV viral suppression. We hypothesized that each format of the intervention would reduce alcohol use compared to the standard of care. We also examined intervention acceptability, uptake, and cost (described elsewhere).

## 2. Methods

### 2.1. Design

This was a 3-arm parallel design RCT with a 1:1:1 allocation ratio.

### 2.2. Setting

The study took place in the semi-urban setting of Mbarara, Uganda. Participants were recruited from the Immune Suppression Syndrome (ISS) Clinic of the Mbarara Regional Referral Hospital (MRRH), an HIV clinic with over 11,000 active patients.

### 2.3. Ethical approval

All study procedures were approved by the institutional review boards of the University of California, San Francisco, the Mbarara University of Science and Technology, and the Uganda National Council on Science and Technology. The study was registered at ClinicalTrials.gov (NCT #03928418) and followed the Consolidated Standards of Reporting Trials (CONSORT) reporting guideline for multi-arm parallel group trial designs (Juszczak et al., 2019).

### 2.4. Inclusion criteria

Inclusion criteria were being infected with HIV, 18 years and older, Alcohol Use Disorders Identification Test – Consumption (AUDIT-C, modified to cover the prior 3 months) positive (≥3 for women, ≥4 for men); having daily access to a working cell phone (confirmed by study staff); being prescribed antiretroviral therapy (ART) for at least six months; living within a 2-hour driving distance or 60 km of the study site; and being fluent in English or Runyankole (the local language). Given the scarcity of mental health professionals in Africa (Sankoh et al., 2018), we included persons reporting unhealthy alcohol use, i.e. the entire spectrum of alcohol use that is harmful to health (Saitz et al., 2021). Persons who planned to move out of the catchment area within six months, were participating in another research study, or were unable to give informed consent, were excluded.

### 2.5. Recruitment

We screened clinic patients who had reported any alcohol use via the AUDIT in their intake clinic visits or by clinic staff knowledge. All participants gave written informed consent to participate in the study in either English or Runyankole.

### 2.6. Randomization and masking

Following baseline questionnaires and blood draw, we randomized participants to: (1) in-person brief workbook-based alcohol counseling at two regularly-scheduled quarterly clinic visits plus interim boosters delivered every 3 weeks by phone (live call arm); (2) in-person brief workbook-based alcohol counseling at two regularly-scheduled quarterly clinic visits plus twice-weekly boosters delivered via two-way automated calls either by SMS or IVR (technology arm); or (3) standard of care which included brief advice, with a wait-listed intervention (control arm). We created a computer-generated randomization list with a 1:1:1 ratio using blocks of nine for pre-printing on scratch cards that the participants scratched with a coin to reveal the randomization arm to themselves and the study staff. There was no masking of study arm. Those randomized to the intervention arms were referred to the study counselor for in-person counseling and training on the booster calls. Those randomized to the control arm had no contact with the study counselors during the first nine months of the study, but were offered the study intervention after their nine-month study visit.

### 2.7. Interventions

The in-person one-on-one counseling sessions, administered by a lay health-counselor in both intervention arms, were conducted in a private room at the clinic using an illustrated workbook. Before the trial, we adapted the workbook from a prior study (Chander et al., 2015) for PWH in Uganda (Leddy et al., 2021). The sessions were originally 20-minutes each, but local communication styles required deprioritizing brevity. The illustrated workbook includes health assessments, discussions of the harms related to drinking, feedback on levels of drinking, discussion of strategies to reduce drinking, and an agreement form for negotiated maximum drinking, that reflected a reduction in the number of drinks per day, days drinking, or both. Participants were given the workbook and a fillable illustrated drinking diary to take home, and were asked to bring a friend or relative to help support them with their drinking goals to their second counseling session. The second session, scheduled to coincide with the participants’ next clinic visit three months later, included reinforcement of the drinking goals and discussion of challenges to reducing drinking, and support person advise on helping the participants with their goals.

Booster sessions, tailored to participants’ drinking agreements and gender, occurred between the two in-person sessions, and were scripted in both intervention arms to check participants’ progress on meeting the drinking agreement, and to provide positive reinforcement when drinking limits were achieved, and encouragement to keep trying when they were not met. The booster calls also allowed for a revision of participants’ drinking goals. Technology arm participants were asked to choose between SMS and IVR for their booster delivery after both were demonstrated by the intervention counselor. The IVR boosters, recorded in the voices of the actual counselors, included simple yes/no responses to recorded questions and were included to account for the expected low literacy of some of the participants. The technology boosters also included the option to request a call back from a live counselor. The live call boosters were delivered every 3 weeks and the technology boosters were delivered twice weekly. Following the first in-person session, the counselors trained the participants in the technology arm on entering a personal identification number to obtain the messages and responding to the booster session questions, with repeated training as needed, and provided a toll-free phone line to contact study staff.

The counsellors were Ugandan graduates of college and the Uganda Ministry of Health HIV counselor training. For this study, they received approximately 10 hours of training and monthly check-ins with a licensed clinical psychologist with experience in Motivational Interviewing (MI) and brief alcohol interventions (SWK). The training included readings on MI, review of the intervention workbook, and multiple structured role-plays of the intervention.

### 2.8. Fidelity

We audio-recorded the in-person counseling sessions and the Ugandan study coordinator reviewed 15 % of them for fidelity (n = 37), using a 17-item check-list of intervention components adapted to the current trial (Pantalon et al., 2012) and assessing the skills of the counsellors in the use of empathy, non-judgmental style, encouragement, warmth, and open-ended questions (Singla et al., 2014).

### 2.9. Adaptations for COVID-19

On March 30, 2020, the Ugandan government issued restrictions on movement that lasted until May 22, 2020. During that time, we ceased in-person study visits. At the start of the restrictions, 31 persons were enrolled and had completed session one only. To avoid in-person contact, we extended delivery of booster sessions, resulting in a mean time between sessions of 3.8 months (95 % CI: 3.6–4.1) for those 31 participants, compared to 2.8 months (95 % CI: 2.8–2.9) in those enrolled after restrictions were lifted.

### 2.10. Assessments

We conducted a structured interviewer-administered questionnaire at baseline, six, and nine months. The baseline questionnaire included demographics and literacy (Morris et al., 2006), and all questionnaires included the 30-day Timeline Follow Back (TLFB)(Sobell and Sobell, 1992), the AUDIT-C (Bradley et al., 2007), adapted to reflect the prior three months, and the Marlowe Crowne social desirability scale (SDS, observed alpha=0.68)(Crowne and Marlowe, 1960). We used pictures of local drinks to define standard drinks. At six months, we also asked the Client Satisfaction Scale (Attkisson and Greenfield, 2004) (observed alpha=0.66) and the System Usability Scale (Bangor et al., 2008) (technology arm only, observed alpha=0.68). We included the Perceived Awareness of the Research Hypothesis scale (Rubin, 2016) (observed alpha=0.41), but due to low reliability we used our own question about the purpose of the research (“To find ways to reduce alcohol use”). At three months, we conducted the TLFB. All questionnaire items were translated into Runyankole and back-translated, and were offered in English and Runyankole. Participants were provided transport refunds, a meal, and a bar of soap at each study visit.

### 2.11. Laboratory testing

We conducted venous blood draws at each study visit. We prepared dried blood spots (DBS) by pipetting 5 μL spots onto Whatman 903 cards. DBS cards were stored at −80 C and shipped in batches to a commercial laboratory for PEth quantification (16:0/18:1 analog), with limit of quantification (LOQ) of 8 ng/mL (Jones et al., 2011). PEth has been shown to decline to undetectable levels after drinking ceases (Gnann et al., 2012), and is well-correlated with the volume of alcohol consumed over the past 2–4 weeks (Ulwelling and Smith, 2018). Due to recent findings of an association of PEth sensitivity with anemia and body mass index (BMI)(Hahn et al., 2021), hemoglobin and BMI were measured at the baseline, six, and nine-month visits. Viral load testing was performed using Gene Xpert at baseline and nine months.

### 2.12. Primary outcomes

The primary outcomes were number of days drinking (NDD) alcohol in the prior 21 (calculated from the 30-day TLFB) and PEth at the six- and nine-month study visits, together, and viral suppression (<40 copies/mL) at nine months. We chose NDD to be consistent with a similar intervention (Chander et al., 2015), and because this measure was most highly correlated with PEth in Uganda (Hahn et al., 2012). In addition, NDD is more reliable than number of standard drinks, which is challenging to measure due to a wide range of drink sizes and concentrations and beverage sharing in Uganda. We chose a 21-day period to be consistent with the window of detection for PEth (Hahn et al., 2016a).

### 2.13. Secondary outcomes

Additional outcomes were the number of heavy drinking days (>3 drinks per day for women and >4 drinks per day for men) in the prior 21 days, the AUDIT-C score (0–12), unhealthy alcohol consumption (AUDIT-C positive), PEth ≥ 50 ng/mL (consistent with unhealthy drinking) (Hahn et al., 2016b), and a composite measure of unhealthy alcohol use (AUDIT-C positive and/or PEth≥50 ng/mL), referred to as AUDIT-C/PEth. We also examined self-reported ART adherence (percent adherent, measured using the lower of a 30-day visual analog scale and the number of days in the prior 30 the participant reported missed pills) and the SDS score. We intended to examine CD4 cell counts extracted from clinic records, but were unable to, because the MRRH ISS Clinic discontinued CD4 testing when viral load monitoring became routine.

### 2.14. Statistical methods

#### 2.14.1. Primary outcomes

We calculated summary statistics (proportions, means, and 95 % confidence intervals [CIs]) at baseline and follow up, overall and by randomization arm. For the primary analyses, we used mixed effects modeling of the follow up time points, using Intention to Treat. The models included random effects for participants, and fixed effects for timepoint (dummy variable for 6 or 9 month), study arm, and the interaction of time and study arm, as well as patient sex and the baseline outcome measurement (i.e. NDD, PEth, or viral suppression). We used the negative binomial distribution for NDD as recommended for alcohol use outcomes (Horton et al., 2007), and linear modeling for log10 transformed PEth (setting transformed values below the LOQ to 0), due to its skewness. In order to directly report adjusted mean values for PEth we estimated a generalized linear model with gamma distribution and log link as is commonly used to model right skewed distributions (Manning and Mullahy, 2001). The generalized linear models yielded the same conclusions as the linear models. We conducted logistic regression for viral suppression at nine months, controlling for sex and baseline viral suppression.

We conducted sensitivity analyses to examine the effect of missing follow-up observations, by setting missing data points to the baseline value. We examined effect modification of the treatment effect for both outcomes by sex, baseline NDD (median split), baseline PEth (median split), self-reported very high-risk alcohol use (baseline AUDIT-C≥8, based on literature showing this cutoff indicated high mean alcohol consumption (Rubinsky et al., 2013)), literacy, and whether each study visit occurred before versus after the implementation of COVID-19 movement restrictions (March 30, 2020), and conducted stratified analyses when interaction was observed (p < 0.100).

#### 2.14.2. Secondary outcomes and post-hoc analyses

We constructed additional models for the secondary outcomes, using the logit link for binary outcomes, negative binomial distribution for count outcomes, and linear models for continuous outcomes, controlling for sex and the baseline level of the outcome variable. Our mixed model for the composite AUDIT-C/PEth variable did not converge; we instead conducted a generalized estimating equation (GEE) logistic regression model. We conducted an analysis of SDS score, to examine whether SDS increased over time in study and whether changes in SDS over time were differential by study arm. To explore discordance between self-report and PEth results, we constructed a model of NDD, including the study arm by follow-up interaction, sex, PEth (log10 ng/mL), SDS score, and the Perceived Awareness of the Research Hypothesis scale as the independent variables. We did not correct for multiple comparisons given guidance against doing so (Juszczak et al., 2019).

We conducted two post-hoc exploratory analyses: 1) adding BMI and anemia to the main model of log10 PEth described above, and 2) excluding the subset of participants with BMI≥ 30 and severe anemia for whom PEth was previously observed to be less sensitive from our main log10 PEth model (Hahn et al., 2021).

#### 2.14.3. Sample size

The a priori sample size calculation assumed 90 % follow-up retention and used a simplifying t-test for power calculations. We found that a sample size of 90 per study arm, chosen for logistical feasibility, would yield 80 % power to detect a difference in the number of drinking days in the prior 21 between two study arms at follow up of ≥ 2.4 days, i.e. nearly 1 fewer day per week, as statistically significant (α = 0.05) assuming that the mean number of drinking days in the prior 21 in the control group at six months would be 7, based on prior data.

## 3. Results

### 3.1. Enrollment and retention

**Fig. 1.**
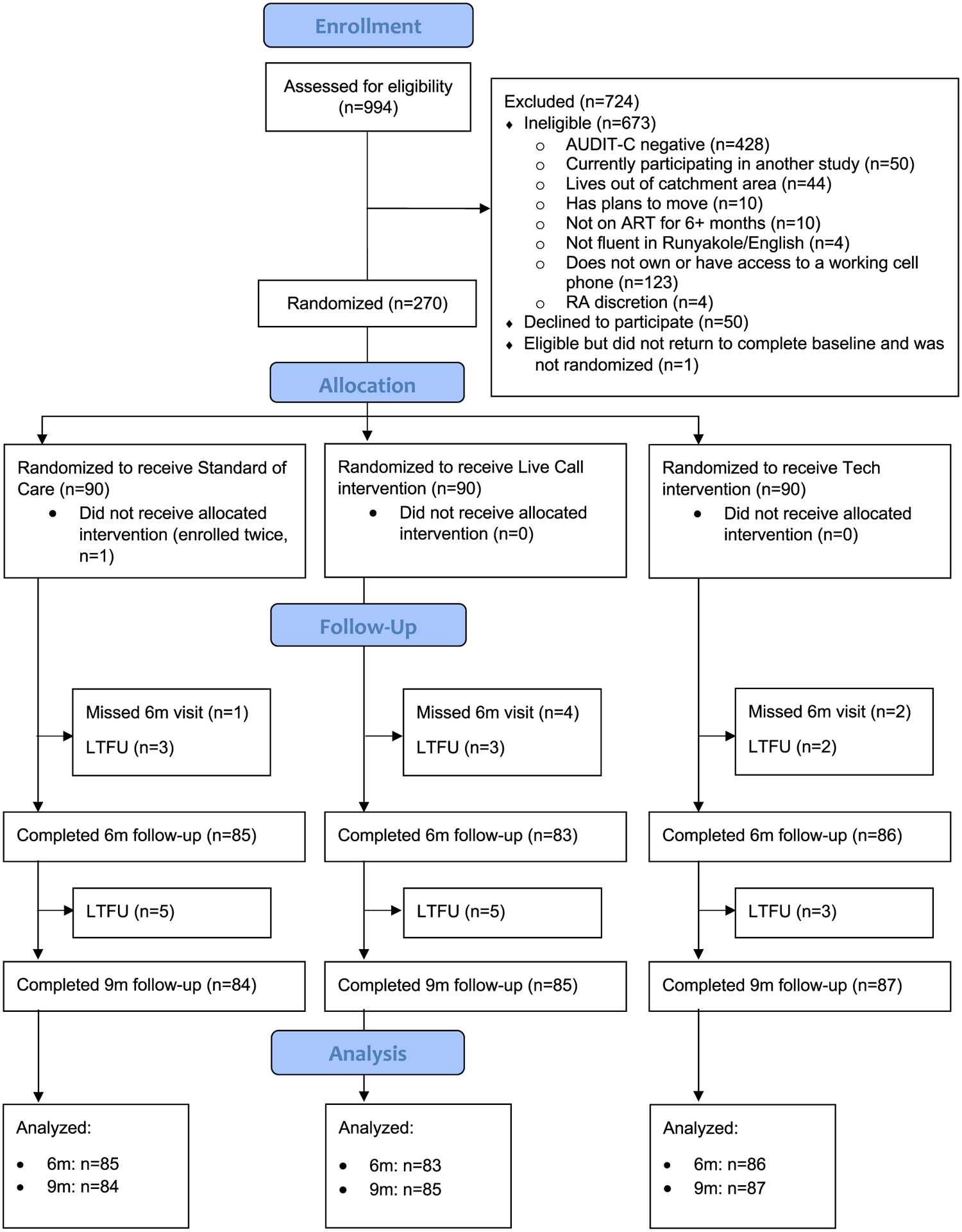
Consort diagram for randomized controlled trial of two brief alcohol interventions versus a control. *See attached.*

From September 19, 2019 through December 20, 2020, 994 persons were approached, 321 were eligible, and 270 were randomized (Fig. 1). After enrollment was completed, one person was found to be enrolled and randomized twice to the control arm; the initial enrollment was retained, so n = 269. Follow-up at six and nine months was 92–94 % in the live call arm, 96–97 % in the technology arm, and 94–96 % in the control arm (Fig. 1). Data were complete for NDDs, but one six-month visit (control arm) was missing a PEth test, and eight nine-month visits (five in the live call arm, three in the technology arm) were missing viral load results due to laboratory failure. There was one adverse event in which a participant reported suicidal thoughts, considered unrelated to study participation, that resolved after referral to a psychiatrist.

Two-thirds (65.4 %) of the participants were male and the mean age was 40.2 (95 % CI: 39.1–41.3)(Table 1). The mean number of self-reported drinking days at baseline was 9.4 (95 % CI: 9.1–9.8), mean PEth was 407.8 ng/mL (95 % CI: 340.7–474.8), and 89.2 % were virally suppressed at baseline.

### 3.2. Intervention adherence and satisfaction

All participants in the intervention arms completed the first in-person session, and 97.8 % of persons in the live counselling arm and 94.4 % of participants in the technology arm completed session 2 (Table 2). The mean length of session 1 was 69.8 min (95 % CI: 68.2–71.4), and the mean length of session 2 was 43.8 min (95 % CI: 41.9–45.7). In the live counselor arm, a mean of 86.5 % (95 % CI: 81.8–91.3) of scheduled booster calls were completed, 44.7 % (95 % CI: 33.4–51.1) of scheduled technology booster sessions were completed. The intervention fidelity and counselling skills were high (>95 % scores), as were satisfaction and usability.

### 3.3. Primary outcomes

**Fig. 2.**
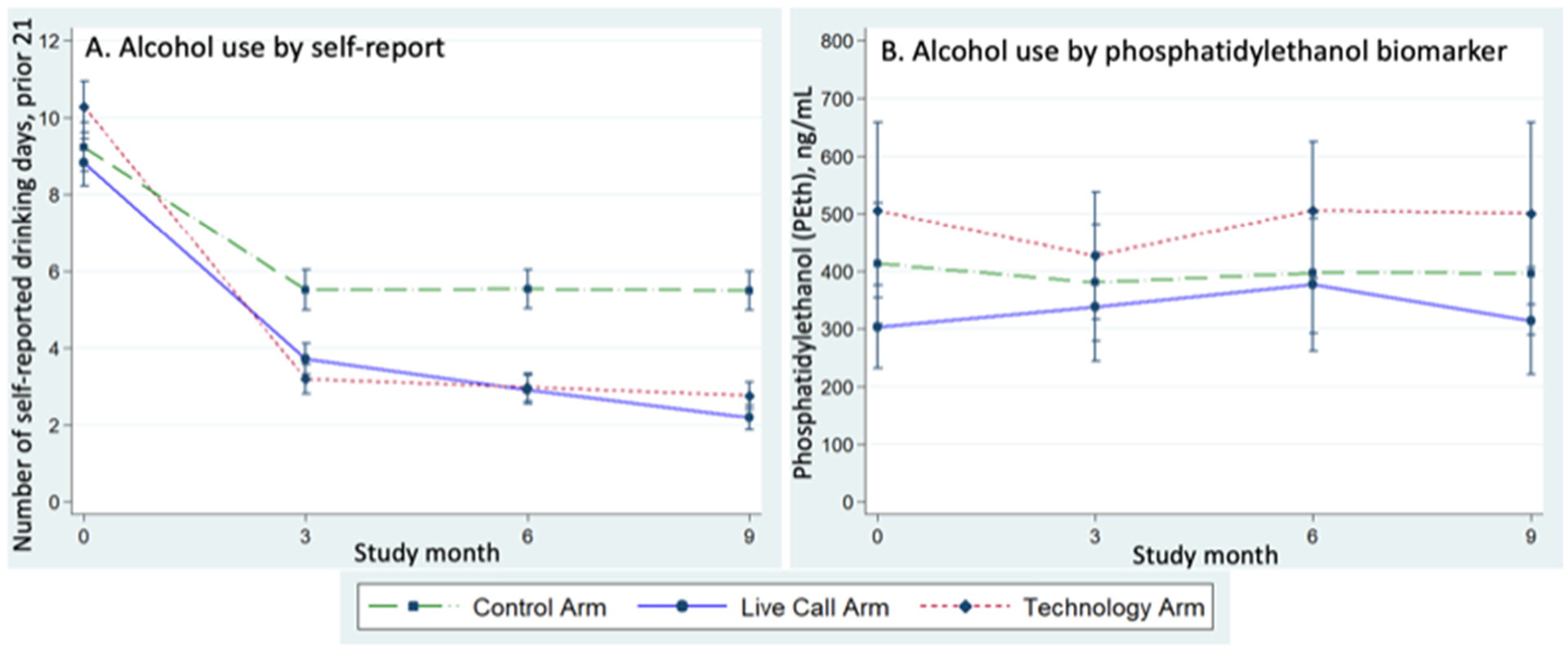
Primary outcomes by study arm over study months. A) Mean self-reported number of drinking days (NDD), prior 21, with 95 % confidence intervals. B) Mean phosphatidylethanol (PEth) ng/mL with 95 % confidence intervals.

The unadjusted means for NDDs and PEth by study month are presented (Fig. 2 and Table 3). We found significant differences in NDDs in the intervention arms at follow-up (six and nine months) compared to the control, i.e. the adjusted mean reduction in NDDs was 3.5 days (95 % CI: 2.1–4.9, p < 0.001) in the live call compared to the control arm and 3.6 days (95 % CI: 2.2–5.1, p < 0.001) in the technology arm compared to the control arm (Table 4). There were no significant differences in follow-up PEth or viral suppression for either intervention arm compared to the control.

We found no evidence of effect modification by sex, baseline alcohol use (above or below the median NDD, PEth, and AUDIT-C≥8), baseline social desirability score (above or below the median), and whether the study visit occurred before or after the COVID-19 restrictions were instituted (p > 0.200, data not shown). There was an interaction of literacy with treatment effects for the NDD outcome (p = 0.05); we found that the treatment effects measured by NDD persisted in both literacy strata, but were stronger in the low literacy group (data not shown). We reached the same conclusions when we included missing data at follow-up as their baseline values in sensitivity analyses (data not shown). The intervention effects on PEth did not differ substantially when we adjusted for BMI and anemia, nor when those with high BMI or anemia (n = 44) were excluded from the analyses (data not shown).

### 3.4. Secondary outcomes and exploratory analyses

The results for secondary alcohol outcome measures were consistent with the main findings. We observed statistically significant decreases in both intervention arms compared to the control at follow up when measured by self-reported number of heavy drinking days, AUDIT-C score, and unhealthy alcohol use by AUDIT-C (Tables 5 and 6). We observed no significant decreases in the proportion with PEth≥ 50 ng/mL in the intervention arms compared to the control. When we used the composite AUDIT-C/PEth variable for unhealthy alcohol use, we found an effect at follow up of the live call arm compared to the control that did not reach statistical significance (p = 0.063). We found no differences in the intervention arms compared to the control in self-reported prior 30-day ART adherence.

SDS appeared to increase over study month (Table 5), however we found no significant differences in SDS between each intervention arm and the control (Table 6). In exploratory analyses, we found an association of SDS with NDD, with a 1-point increase in SDS associated with an 3 % decrease in the reported NDD, but this did not reach statistical significance (p = 0.067, data not shown). Awareness of the study aim to reduce alcohol use was high (71.1 %) and not associated with NDDs (data not shown).

## 4. Discussion

We compared two versions of a brief multi-session alcohol intervention targeted to PWH with unhealthy alcohol use adapted for the Ugandan context (Leddy et al., 2021) to a control condition, and found significant intervention effects when measured by self-reported alcohol use, but no effects when measured by PEth, a well validated alcohol biomarker (Gnann et al., 2012). The mean PEth follow-up levels were well above 200 ng/mL, a cutoff for chronic excessive alcohol use (Luginbühl et al., 2022). Thus, we conclude that there was no significant intervention effect on alcohol use despite the self-reported effect. We also failed to find effects on viral suppression. This study occurred concurrently with the start of the COVID-19 pandemic, which was a stressful time; we found in another study that alcohol consumption increased in PWH in southwest Uganda, despite government-ordered bar closures (Asiimwe et al., 2022). It is plausible that the effects of the pandemic overwhelmed any intervention effects that might have otherwise occurred.

We suspect that the self-reported decreases in alcohol use were at least in part due to social desirability, as SDS scores increased over the study period. In qualitative interviews conducted after the intervention (manuscript under review), participants reported feeling personally cared for by the study staff and counselors, motivated to reduce their alcohol use, and feeling shame when staff asked them whether they had reduced their drinking if they had not. The differences in results when measured by self-report versus PEth are consistent with discrepancies found in observational studies of PWH in Uganda (Adong et al., 2019; Hahn et al., 2016b; Magidson et al., 2019; Muyindike et al., 2017) and in intervention trials among PWH in Kenya (Papas et al., 2016) and the USA (McGinnis et al., 2021).

Prior trials of the efficacy of counselling-based alcohol interventions for PWH on alcohol use have shown either small or inconclusive intervention efficacy (Madhombiro et al., 2019; Scott-Sheldon et al., 2017); results of studies conducted among PWH in sub-Saharan Africa have also been mixed (Huis In ‘t Veld et al., 2019; Kane et al., 2022; Madhombiro et al., 2020; Magidson et al., 2021; Papas et al., 2021; Wandera et al., 2016). The previous findings are based on self-report; to dispel uncertainty, we recommend that future alcohol intervention studies among PWH use PEth alone or combined with self-report as the primary outcome.

The primary strengths of this study are that we measured PEth, and we had high follow-up rates. We were limited in our ability to study the impact of the interventions on viral suppression because viral non-suppression, which is low in Uganda (10 %) (Lecher et al., 2021) was not an eligibility criterion, and the study power calculations were not based on this outcome. In addition, the high proportion with viral suppression suggests that improving HIV health may not have been a motivator for reducing alcohol use. We were also limited in our ability to prevent contamination across study arms; however, we felt the likelihood of this was low because the intervention occurred at a large HIV clinic (with approximately 150 patients seen per day). Our findings may have been limited by not excluding persons with severe alcohol use disorder; however, the lack of effect modification by high-risk alcohol use at baseline suggests this was not a major factor. The single site and the occurrence of a global pandemic limit the generalizability of our findings.

In summary, while we found effects on self-reported alcohol use, we found no effects on PEth, indicating that neither version of this brief intervention was sufficient to substantially impact alcohol use. Because unhealthy alcohol use causes multiple co-morbidities to PWH (Williams et al., 2016), there is urgent need for further work to reduce alcohol use among PWH. We urge further work to develop effective screening and interventions that can be feasibly integrated into HIV and other chronic disease care. Other interventions should also be examined, including the use of pharmacologic methods, transdiagnostic interventions that target the underlying processes that drive unhealthy alcohol use, and incentive-based interventions, with attention paid to the feasibility of implementation in low-resource settings. In HIV care, future interventions should focus on those populations at highest risk, such as those missing clinic visits or not attaining viral suppression. The discrepancy we observed between self-report and PEth also highlights the urgent need to use objective measures as primary outcomes in future behavioral trials in PWH.

## Figures and Tables

**Table 1:** Baseline characteristics, overall and by study arm.

	Overall (n = 269)	Control arm (n = 89)	Live call arm (n = 90)	Technology arm (n = 90)
	n (%)	n (%)	n (%)	n (%)
Sex				
Female	93 (34.6 %)	30 (33.7 %)	33 (36.7 %)	30 (33.3 %)
Male	176 (65.4 %)	59 (66.3 %)	57 (63.3 %)	60 (66.7 %)
Literate/somewhat literate				
No	37 (13.8 %)	15 (17.1 %)	9 (10.0 %)	13 (14.4 %)
Yes	231 (86.2 %)	73 (83.0 %)	81 (90.0 %)	77 (85.6 %)
Virally suppressed				
No	29 (10.8 %)	8 (9.0 %)	13 (14.4 %)	8 (8.9 %)
Yes (<40 copies/mm^3^)	240 (89.2 %)	81 (91.0 %)	77 (85.6 %)	82 (91.1 %)
	**Mean (95 % CI)**	**Mean (95 % CI)**	**Mean (95 % CI)**	**Mean (95 % CI)**
Age	50.2 (39.1−41.3)	40.2 (38.1−42.3)	40.7 (38.6−42.7)	39.8 (37.9−41.6)
Years since HIV diagnosis (n = 248)	8.9 (8.2−9.6)	9.3 (8.1−10.4)	9.3 (8.0−10.6)	8.2 (7.1−9.2)
Years since ART initiation (n = 240)	7.3 (6.7−7.9)	7.8 (6.8−8.9)	7.1 (5.9−8.2)	7.0 (6.1−7.9)
Social desirability score	18.4 (18.0−18.8)	18.2 (17.5−18.8)	18.1 (17.4−18.8)	18.9 (18.2−19.6)
Number of drinking days, past 21	9.4 (9.1−9.8)	9.2 (8.6−9.9)	8.8 (8.2−9.5)	10.3 (9.6−11.0)
PEth (ng/mL)	407.8 (340.7−474.8)	413.3 (306.6−519.9)	303.9 (230.7−377.1)	506.2 (351.8−660.5)

**Table 2:** Intervention uptake and satisfaction, overall and by intervention arm.

	Overall	Control	Live Call	Technology
	(n = 269)	(n = 89)	(n = 90)	(n = 90)
**Counseling sessions**				
% completed in-person session 1	100.0 %	-	100.0 %	100.0 %
Length of session 1 (minutes), mean (95 % CI)	69.8 (68.2−71.4)	-	69.9 (67.3−72.6)	69.6 (67.7−71.6)
% completed in-person session 2	96.1 %	-	97.8 %	94.4 %
% brought helper to session 2	39.3 %	-	43.2 %	35.3 %
Length of session 2 (minutes), mean (95 % CI)	43.8 (41.9−45.7)	-	45.2 (41.8−48.6)	42.3 (40.6−44.0)
Fidelity checklist %, mean (95 % CI)	100.0 % (100.0−100.0) (n = 37)	-	100.0 % (100.0−100.0) (n = 18)	100.0 % (100.0−100.0) (n = 19)
Counseling skills %, mean (95 % CI)	95.7 % (94.2−97.3) (n = 37)	-	94.0 % (91.4−96.6) (n = 18)	97.4 % (95.7−99.0) (n = 19)
**Booster sessions**				
# attempted per participant, mean (95 % CI)	-	-	3.2 (3.1−3.4)	23.5 (22.3−24.7)
% completed per participant, mean (95 % CI)	-		86.5 % (81.8−91.3)	44.7 % (33.4−51.1)
Length of booster calls (minutes), mean (95 % CI)	-	-	6.4 (6.2−6.6)	-
**Intervention satisfaction**				
Client Satisfaction Questionnaire – 8, mean (95 % CI)	92.1 (91.2−93.0) (n = 254)	90.0 (88.2−91.8) (n = 85)	93.3 (91.9−94.8) (n = 83)	93.1 (91.8−94.5) (n = 86)
System Usability Scale, mean (95 % CI)	-	-	-	34.9 (34.2−35.5) (n = 86)
Thought the researchers were studying:	(n = 254)	(n = 85)	(n = 83)	(n = 86)
Ways to quit/reduce alcohol use	71.3 %	67.1 %	66.3 %	80.2 %
Ways to improve health	22.1 %	23.5 %	26.5 %	16.3 %
Ways to improve taking medications	6.7 %	9.4 %	7.2 %	3.5 %

**Table 3:** Primary outcomes by study arm for each study visit, unadjusted results.

	Control arm	Live call arm	Technology arm
**Number of drinking days, prior 21, mean (95 % CI)**			
Baseline	9.2 (8.6–9.9)	8.8 (8.2–9.5)	10.3 (9.6–11.0)
3–month	5.5 (5.0–6.1)	3.7 (3.3–4.2)	3.2 (2.8–3.6)
6-month	5.5 (5.0–6.1)	2.9 (2.6–3.3)	3.0 (2.6–3.4)
9-month	5.5 (5.0–6.0)	2.2 (1.9–2.5)	2.8 (2.4–3.1)
**PEth ng/mL, mean (95 % CI)**			
Baseline	413.3 (306.6–519.9)	303.9 (230.7–377.1)	506.2 (351.8–660.5)
3-month	380.5 (278.0–483.1)	338.0 (242.8–433.1)	427.0 (314.7–539.4)
6-month	396.9 (290.5–503.4)	376.9 (259.6–494.1)	506.2 (386.1–626.3)
9-month	396.2 (288.1–504.3)	313.8 (220.3–407.4)	500.8 (340.2–661.3)
**Viral suppression, % (95 % CI)**			
Baseline	91.0 % (83.1–96.0)	85.6 % (76.6–92.1)	91.1 % (83.2–96.1)
9-month	92.9 % (85.1–97.3)	95.0 % (87.7–98.6)	94.1 % (86.7–98.0)

Viral load tested at baseline and nine months.

**Table 4:** Estimated means and 95 % confidence intervals at follow up, difference in means compared to the control, and p-values for primary outcomes from regression models adjusted for sex and the baseline value of each outcome.

	Control	Live call	Technology
**Number of drinking days, prior 21**			
Mean (95 % CI)	6.2 (4.8–7.5)	2.7 (2.0–3.3)	2.5 (2.0–3.1)
Control minus Intervention arm (95 % CI)	-	3.5 (2.1–4.9)	3.6 (2.2–5.1)
p-value	-	< 0.001	< 0.001
**PEth (ng/mL)**			
Mean (95 % CI)	528.6 (397.3–659.9)	492.2 (369.1–615.3)	559.5 (426.7–692.3)
Control minus Intervention arm (95 % CI)	-	36.4 (−117.5 to 190.3)	−30.9 (−194.8 to 132.9)
p-value	-	0.643	0.711
**Viral suppression**			
% (95 % CI)	92.9 % (87.5–98.2)	95.2 % (90.7–99.7)	93.8 % (88.7–98.9)
Control minus Intervention arm (95 % CI)	-	−2.3 % (−9.3 to 4.7)	−0.9 % (−8.3 to 6.4)
p-value	-	0.515	0.801

**Table 5:** Secondary outcomes by study month for each month, unadjusted results.

	Control arm	Live call arm	Technology arm
**Number of heavy drinking days (> 3 drinks per day for women and > 4 drinks per day for men) prior 21, mean (95 % CI)**			
Baseline	6.0 (5.5–6.5)	4.9 (4.4–5.4)	5.7 (5.2–6.2)
3-month	2.8 (2.5–3.2)	0.9 (0.7–1.1)	1.5 (1.2–1.8)
6-month	3.6 (3.2–4.0)	1.2 (1.0–1.5)	1.4 (1.1–1.7)
9-month	3.3 (2.9–3.7)	0.6 (0.5–0.8)	1.5 (1.3–1.8)
**AUDIT-C (prior 3 months) score (0–12), mean (95 % CI)**			
Baseline	7.1 (6.5–7.7)	6.8 (6.2–7.4)	6.9 (6.3–7.5)
6-month	5.6 (4.8–6.4)	3.1 (2.5–3.7)	3.5 (2.8–4.1)
9-month	5.3 (4.6–6.1)	2.7 (2.2–3.3)	2.9 (2.2–3.5)
**Unhealthy alcohol use via AUDIT-C** * **, % (95 % CI)**			
Baseline	95.5 % (88.8–98.7)	96.7 % (90.6–99.3)	97.8 % (92.2–99.7)
6-month	66.7 % (55.5–76.6)	43.4 % (32.5–54.7)	46.5 % (35.7–57.6)
9-month	69.0 % (58.0–78.7)	32.9 % (23.1–44.0)	40.2 % (29.9–51.3)
**PEth ≥ 50 ng/mL, % (95 % CI)**					
Baseline	77.5 % (67.4–85.7)	77.8 % (67.8–85.9)	86.7 % (77.9–92.9)
3-month	74.0 % (62.8–83.4)	73.9 % (63.4–82.7)	81.2 % (71.2–88.8)
6-month	78.6 % (68.3–86.8)	72.3 % (61.4–81.6)	83.7 % (74.2–90.8)
9-month	78.6 % (68.3–86.8)	72.9 % (62.2–82.0)	86.2 % (77.1–92.7)
Unhealthy alcohol use via AUDIT-C* **and/or PEth ≥ 50 ng/mL, % (95 % CI)**			
Baseline	100.0 % (95.9–100.0)	97.8 % (92.2–99.7)	98.9 % (94.0–100.0)
6-month	90.6 % (82.3–95.8)	78.3 % (67.9–86.6)	89.5 % (81.1–95.1)
9-month	88.1 % (79.2–94.1)	78.8 % (68.6–86.9)	87.4 % (78.5–93.5)
**ART adherence, mean % (95 % CI)**			
Baseline	88.7 % (86.0–91.4)	90.0 % (87.2–92.8)	86.4 % (82.2–90.7)
6-month	90.4 % (88.2–92.7)	92.3 % (89.4–95.2)	91.6 % (88.4–94.7)
9-month	92.8 % (91.0–94.5)	93.3 % (91.3–95.4)	93.3 % (90.3–96.2)
**Social Desirability Score (SDS), mean (95 % CI)**			
Baseline	18.2 (17.5–18.8)	18.1 (17.4–18.8)	18.9 (18.2–19.6)
6-month	19.1 (18.4–19.8)	19.0 (18.3–19.8)	19.5 (18.7–20.3)
9-month	21.3 (20.6–22.0)	21.2 (20.5–21.9)	22.0 (21.3–22.7)

* Prior 3 months, ≥ 3 for women, ≥ 4 for men

AUDIT-C and SDS, and ART adherence measured at baseline, six, and nine months.

**Table 6:** Estimated means and 95 % confidence intervals at follow up, difference in means compared to the control, and p-values for secondary outcomes from regression models adjusted for sex and the baseline value of each outcome.

	Control	Live call	Technology
**Number of heavy drinking days (> 3 drinks per day for women and > 4 drinks per day for men) prior 21**			
Mean (95 % CI)	5.8 (2.8–8.9)	1.4 (0.6–2.1)	1.5 (0.7–2.3)
Control minus Intervention arm (95 % CI)	-	4.4 (1.6–7.3)	4.3 (1.5–7.2)
p-value	-	0.002	0.003
**AUDIT-C (prior 3 months) score (0–12)**					
Mean (95 % CI)	5.4 (4.8–5.9)	3.0 (2.5–3.5)	3.2 (2.7–3.7)
Control minus Intervention arm (95 % CI)	-	2.3 (1.6–3.0)	2.2 (1.5–2.9)
p-value	-	< 0.001	< 0.001
**Unhealthy alcohol use via AUDIT-C** *					
% (95 % CI)	67.7 % (59.8–75.7)	38.8 % (30.1–47.5)	42.8 % (34.0–51.6)
Control minus Intervention arm (95 % CI)	-	28.9 % (17.0–40.7)	24.9 % (13.0–36.8)
p-value	-	< 0.001	< 0.001
**PEth ≥ 50 ng/mL**			
% (95 % CI)	77.1 % (70.8–83.4)	72.8 % (66.2–79.4)	81.1 % (74.7–87.4)
Control minus Intervention arm (95 % CI)	-	4.3 % (−4.0 to 12.6)	−4.0 % (−12.1 to 4.2)
p-value	-	0.306	0.337
Unhealthy alcohol use via AUDIT-C* **and/or PEth ≥ 50 ng/mL (%)**			
% (95 % CI)	88.4 % (82.7–94.1)	79.7 % (72.5–86.9)	88.5 % (82.7–94.3)
Control minus Intervention arm (95 % CI)	-	8.7 % (−0.5 to 18.0)	−0.1 % (−8.2 to 8.0)
p-value	-	0.063	0.980
**ART adherence %**			
Mean (95 % CI)	91.6 (89.5–93.7)	92.4 (90.3–94.5)	92.7 (90.6–94.8)
Control minus Intervention arm (95 % CI)	-	−0.8 (−3.8 to 2.2)	−1.1 (−4.1 to 1.9)
p-value	-	0.610	0.474
**Social Desirability Score (SDS)**					
Mean (95 % CI)	20.3 (19.7–20.8)	20.2 (19.7–20.8)	20.6 (20.0–21.1)
Control minus Intervention arm (95 % CI)	-	0.0 (−0.7 to 0.8)	−0.3 (−1.0 to 0.5)
p-value	-	0.946	0.454

* Prior 3 months, > 3 for women, > 4 for men

## Abbreviations:

PEth: Phosphatidylethanol
PWH: Persons with HIV
